# Effect of Obesity on Neoadjuvant Systemic Therapy Outcomes in Patients with Early Breast Cancer: A Retrospective Institutional Study

**DOI:** 10.31557/APJCP.2020.21.3.683

**Published:** 2020-03

**Authors:** Ayman Rasmy, Yasser Sorour

**Affiliations:** 1 *Medical Oncology, Zagazig University, Zagazig, *; 4 * Department of Radiotherapy, National Cancer Institute, Cairo University, Cairo, Egypt, *; 2 *Medical Oncology, King Saud Medical City, Riyadh, *; 3 * Department of Adult Oncology, Oncology Center, King Fahd Specialist Hospital, Saudi Arabia. *

**Keywords:** Obesity BMI, early breast cancer, neoadjuvant therapypCR, PFS, OS

## Abstract

**Background:**

Obesity and overweight are usually considered as poor prognostic factors in early breast cancer. Body mass index (BMI) is a significant predictive factor for lower pathologic complete response (pCR) rates after neo-adjuvant systemic therapy (NST). The relationship between obesity and breast cancer prognosis varies according to patient and tumor characteristics such as menopausal status and tumor subtype, respectively.

**Patients and Methods:**

Between March 2010 and October 2013, 80 patients with early breast cancer who had received standard NST from KFSH Saudi Arabia were included in this study. For statistical analysis, the study participants were categorized into two groups based on their BMI, as normal (BMI < 25 kg/m^2^) and obese groups (BMI ≥ 25 kg/m^2^). pCR was defined as non-invasive cancer in the breast/axillary tissue.

**Results:**

The median age of our patients was 48 (range, 38-68) years. Invasive ductal carcinoma (IDC) subtype was identified in 93.8% of the cases. Additionally, 26 (32.5%) and 33 (41.25%) patients were diagnosed with stage II and stage IIIA breast cancer, respectively. Lymphovascular invasion was detected in 32.5%, whereas intermediate and high-grade malignancy were found in 61.25% and 32.5% of the patients, respectively. Forty-four patients (55%) were obese. pCR was achieved in 56 patients (70%), and the comparison between patients with and without pCR revealed that those in the former group had significantly lower tumor grades. Significantly, lower relapse and mortality rates were distinguished in patients who achieved pCR than in those who did not. Additionally, comparison between normal and obese patients revealed that a high number of patients in both groups were post-menopausal (p = 0.001). However, survival analysis indicated the absence of significant differences in disease-free survival between the two groups based on BMI (p = 0.19). Conversely, patients with normal BMI had significantly better overall survival than obese patients (p = 0.029), with a higher mortality rate noted in the obese group (16.7% vs 2.3%, p = 0.037).

**Conclusions:**

In the present study, 58.3% of patients that failed to achieve pCR had BMI above the normal level; they moreover had higher relapse rates and lower survival compared with normal BMI patients. This finding needs to be verified through further prospective studies to determine if BMI is a risk factor for breast cancer.

## Introduction

The prevalence of obesity/being overweight and the accompanying cancer incidence has increased over the past few decades. Between 1975 and 2016, the prevalence of increasing body weight in adults “defined as a body mass index of more than 25” increased from approximately 21% in men and 24% in females to approximately 40% in both genders. This increasing trend and parallel population explosion are accompanied by more than 6-fold increase in the number of obese adults from 100 to 671 million (Sung et al., 2019).

Each 5-unit increase in body mass index (BMI) is associated with an approximately 12% increased risk of postmenopausal breast cancer, and the risk is higher in Asians (37%) compared with North Americans and Europeans (10%) (Sung et al., 2019).

Breast cancer is the most common malignancy in females and an important cause of mortality. Several meta-analyses have shown that obesity is a prognostic factor for poorer outcomes after breast cancer diagnosis. In the largest of these studies, Chan et al., (2014) described one meta-analysis of 82 clinical researchers comprising “213075 breast cancer survivors.” A pre-diagnosis BMI ≥30 kg/m^2^ was accompanied by a mortality risk of 1.41 (95% CI: 1.29-1.53) compared with normal weight patients (BMI, 20–24.9); the relative risk for BMI between 25–29.9 was also significantly increased at 1.07 (95% CI: 1.02-1.12) (Momenimovahed and Salehiniya, 2019).

Chan et al., (2014) suggested that obesity at the time of diagnosis of early (curable) breast cancer is accompanied with a low survival rate in both pre- and postmenopausal females. Iwase et al., (2016) mentioned that; “to assess a patient’s fat distribution, various measures including abdominal circumference (AC), subcutaneous fat area (SFA), visceral fat area (VFA), and skeletal muscle area (SMA) are often recognized”.

Obesity is anticipated to decrease the response to neoadjuvant systemic therapy (NST) in breast cancer patients, but the association between actual body composition and NAT outcomes remains unclear (Iwase et al., 2016). Breast cancer studies have reported that “obesity is associated with lower overall survival OS.” As the standard NAT regimen improved after 2000, this clinical benefit could overcome the difficulty of achieving pCR in obese patients (Kogawa et al., 2018).

Obesity stimulates cancer progression by “enhancing cell proliferation, cell survival, invasion/metastasis, and angiogenesis.” These effects are usually arbitrated by “the initiation of insulin resistance, and inflammation and increase in leptin, adiponectin, plasminogen activator inhibitor-1, and vascular endothelial growth factor levels” (van Kruijsdijk et al., 2009).

Additionally, obesity has become recognized as a “chronic pro-inflammatory state”. Consequently, cytokines secreted by adipose tissue can activate macrophages, and in obese persons, adipose tissue is infiltrated with macrophages. Thus, it has been validated that “tumor-associated macrophages have a key role in the tumor microenvironment.” Increased macrophage chemo-attractant protein-1 in breast cancer extracts is an early predictor of early relapse and metastasis. Hence, proliferating macrophages in breast cancer are similarly accompanied with a high tumor grade and poor outcomes (Heetun et al., 2017). 

BMI is a simple surrogate marker of obesity; however, it is not a reliable measure of body fat as it can neither distinguish between lean and fat mass, nor characterize body fat distribution (Heetun et al., 2017). 

To explain the association between poor prognosis and obese breast cancer patients with hormone receptor (HR) positive disease, one theory suggests that high adipose tissue mass might lead to “increased aromatase enzyme activity and estradiol levels,” that ultimately results in increased bioavailability of estrogen, favoring tumor development and evolution when tumors express estrogen receptors (ERs) (de Azambuja et al., 2010). Most researchers consider that higher BMI is correlated with “higher circulating concentrations of sex hormones,” mainly estradiol, insulin, and insulin-like growth factor (ILGF), which leads to an alteration of the normal balance between “cell discrepancy and apoptosis, progression, and proliferation of breast cancer cells” (Nahleh, 2011).

In post-menopausal females, a unique source of estrogen is fatty tissue, in which, the transformation of androgens to estrogens by aromatase enzyme action occurs. Estrogen facilitates the progression of breast malignancy via the estrogen receptor and by the cross talk between estrogen and ILGF pathways (Rose et al., 2004). 

Most important research performed with 1169 patients on the association between chemo-sensitivity and obesity in breast cancer at the MD Anderson Cancer Center in 2008, revealed that “the pCR rate was significantly lower in overweight/obese patients with BMI ≥ 25 kg/m^2^ than in normal/underweight patients with BMI < 25 kg/m^2^” (Litton et al., 2008).

## Materials and Methods

In the current retrospective study, 80 patients that were pathologically and radiologically confirmed with unilateral early breast cancer (stage I = 26.2%, stage II = 32.5%, and IIIA = 41.2%) were enrolled between March 2010 and October 2013 from King Fahad Hospitals, Saudi Arabia. The inclusion criteria were as follows: patients confirmed with unilateral early breast cancer according to the National Cancer Institute definition (early-stage breast cancer is the breast cancer that has not spread beyond the breast or the axillary lymph nodes). This includes stage I, stage IIA, stage IIB, and stage IIIA breast cancers, performance status of 0−2 according to ECOG (Eastern Cooperative Oncology Group). Pathologically confirmed by core needle biopsy samples from all patients at time of initial diagnosis (with/without axillary lymph node FNA evaluation) were examined by microscopic examination and immune stain for “histological type, grade, and hormone status subtype as well as HER2 status.” 

Standard neo-adjuvant chemotherapy was administered to all patients as follows: anthracycline-based combinations in the form of AC (Doxorubicin plus Cyclophosphamide) for 4 cycles or FEC100 for 3 cycles (Fluorouracil–Epirubicin–Cyclophosphamide), followed by Taxens in form of Docetaxel (3-4 cycles every 3 weeks) or paclitaxel (3-4 cycles every 3 weeks or weekly for 12 weeks) with or without Trastuzumab for HER2 Positive patients with Filgrastim support if indicated. Carboplatin and Taxane (CbT) was the protocol used in triple-negative cases. Patients with relapse received subsequent treatment as per standard guidelines. All patients enrolled in the study underwent breast surgery within four weeks of the chemotherapy completion, which was either breast-conserving surgery or mastectomy, as determined by the breast surgeon.

Adjuvant endocrine therapy was provided to patients who were hormone receptor positive (ER-positive and/or PR positive) and was accompanied by adjuvant radiotherapy if indicated. HER2-positive patients received 17 cycles of Trastuzumab. The pCR for the breast and axilla was defined as “the complete disappearing of all invasive malignant cells from breast tissue and regional lymph nodes, regardless of the presence of residual ductal carcinoma in situ” (Green et al., 2005).

BMI is calculated using the formula BMI = kg/m^2^, where kg is a patient’s weight in kilograms and m^2^ is their height in meters squared. Four groups were created based on BMI status, following the World Health Organization recommendations: Group 1: Underweight (BMI < 18.5 kg/m^2^); Group 2: Normal weight (18.5 ≥ BMI < 25 kg/m^2^); Group 3: Overweight (25 ≥ BMI < 30 kg/m^2^); and Group 4: Obese (BMI ≥ 30 kg/m^2^) (Kogawa et al., 2016).

For all the enrolled patients, the weight and height documented during the first treatment cycle were used for BMI calculation. Patients were categorized as normal, overweight, and obese; underweight patients were excluded. For statistical analysis, the patients were grouped into two groups: Group 1 for patients with normal BMI (18.5 ≥ BMI < 25 kg/m^2^), i.e., normal patients, and Group 2 for patients with high BMI (all patients with BMI ≥25), i.e., obese patients (obese and overweight).

*Statistical Analysis*

The reported data were statistically analyzed using SPSS 25. Categorical variables were expressed as number and percent. Variables were compared using the Chi-square test or Fisher’s exact test as appropriate. Kaplan Meyer survival function was used to assess disease-free survival (DFS) and overall survival (OS). Log-rank test was used to detect the effect of co-variables on survival. P value less than 0.05 was considered statistically significant. Cox regression hazard analysis was used to discover significant predictors of survival. Significant variable in univariate regression analysis were advanced to multivariate analysis provided no multicollinearity was detected among variables. P value less than 0.05 was considered statistically significant.

The relationship between the BMI and clinic-pathologic parameters was assessed using the Spearman rank order correlation or Mann-Whitney U test as appropriate.

Overall survival defined as “the time from randomization to death from any cause,” is a direct measure of clinical benefit to a patient. Patients alive or lost to follow-up are censored. As an endpoint, OS is easily stately, unmistakable, objective, touched to be clinically significant, and unaffected by the timing of assessment. Progression-free survival (PFS) was defined as “the time from randomization until the first evidence of tumor progression or until death from any cause, whichever comes first.” Patients that did not die or experience disease progression and those lost to follow-up were excluded from the analysis. By definition, PFS occurs more rapidly and more frequently than OS. Therefore, PFS data are accessible much earlier than OS data (Cheema and Burkes, 2013). 

## Results

Eighty patients were included in this retrospective study; they were classified as stage I (n = 21; 26.25%), stage II (n = 26; 32.5%), and stage IIIA (n = 33; 41.25%). Eighty percent of our patients were postmenopausal. Neo-adjuvant chemotherapy was appropriate for all patients. The mean age was 50.8 ± 7.8 years, and the median follow up period was 49.0 (46.0 – 53.8) months. 

In our study, 56 patients (70.0 %) achieved pathological complete response (pCR). Additionally, pathological analyses revealed that 93.8% and 6.2% of the patients were affected by invasive ductal carcinoma and invasive lobular carcinoma, respectively. Lympho-vascular invasion was observed in 26 patients (32.5%), and 61.25% of the breast cancers showed intermediate grade features. The clinical data of the studied patients are shown in Table 1. 

**Figure 1:**
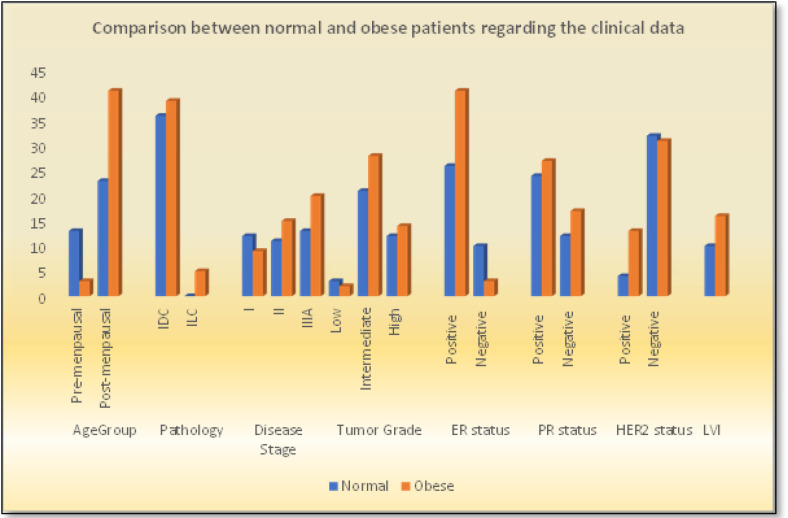
Comparison between Normal and Obese Patients Regarding the Clinical Data

**Figure 2:**
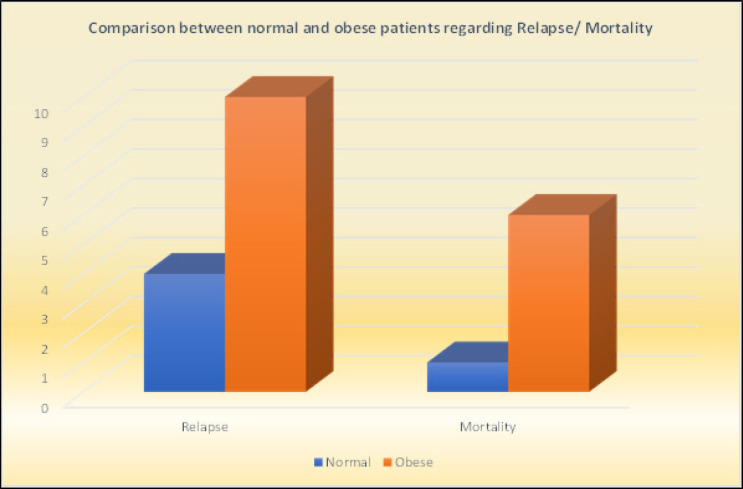
Comparison between Normal and Obese Patients Regarding Relapse/ Mortality

When BMI was examined with respect to menopausal status, a higher number of obese patients were found in the menopausal group, and the correlation was statistically significant (p = 0.001). Statistically significant associations were also found between BMI and pathological subtype (p = 0.001), ER status (p = 0.011), and HER2 status (p = 0.045) (Table-2, Figure 1). Furthermore, normal BMI group experienced lower relapse rate (11.1 % Vs 22.7%., p = 0.16), and lower mortality rates than overweight/ obese group (2.3 % Vs 16.7 %., p = 0.023), (Table 2, Figure 2).

**Figure 3.**
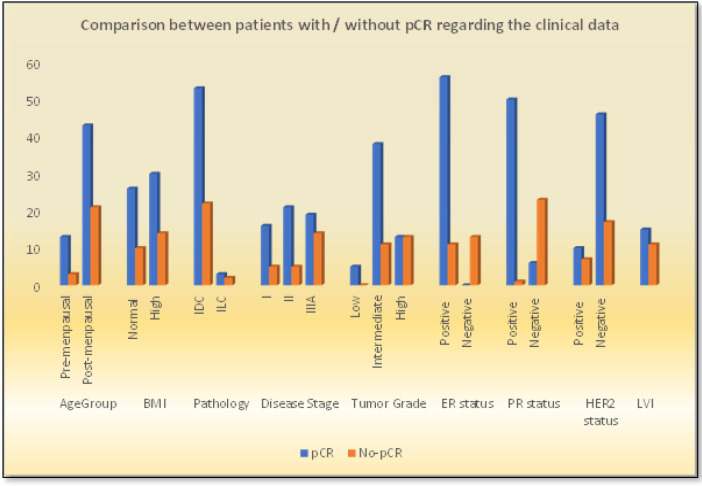
Comparison between Patients with / without pCR Regarding the Clinical Data

**Figure 4:**
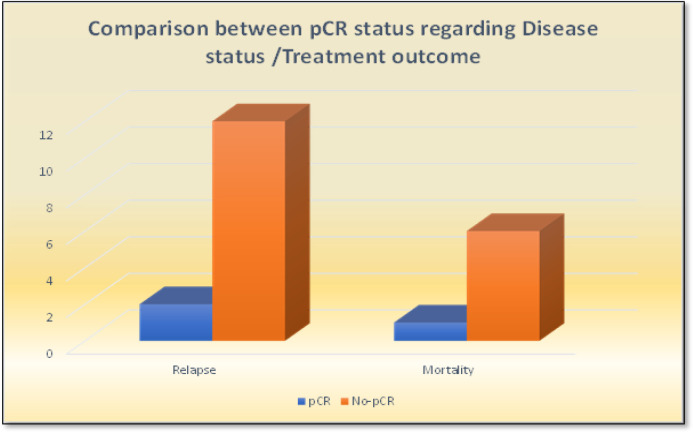
Comparison between pCR Status Regarding Relapse/ Mortality

Pathological complete response (pCR) was achieved in 56 patients (70%), as shown in Table 3. Comparison between patients with and without pCR revealed that patients in the former group had significantly lower tumor grades (Figure 3). They also experienced significantly lower relapse and mortality rates than those without pCR (Figure 4).

Moreover, 30 of the 56 (60.4%) patients who achieved pCR had high BMI, while the remaining 26 (39.6%) patients had normal BMI. Conversely, the correlation between pCR and BMI was not statistically significant (p = 0.7). Similarly, no statistically significant association between pCR and HER2 status was noted (p = 0.2), but ER status showed statistically significant correlation with pCR (p = 0.03). In contrast, significantly higher Ki67 expression was recorded in patients who failed to achieve pCR (p < 0.001) (Figure 3). 

The mortality rate in the studied patients was 8.8 %, with a median (IQR) survival of 35.0 (34.0-36.0) months; median survival of 36 and 31 months was recorded for those that did and did not attain pCR, respectively. Moreover, patients without pCR were statistically more likely to relapse (12 vs. 2 for those with pCR, p < 0.001). Finally, a higher mortality rate was recorded in patients who failed to achieve pCR (p = 0.001) (Table 3, Figure 4). 

**Figure 5:**
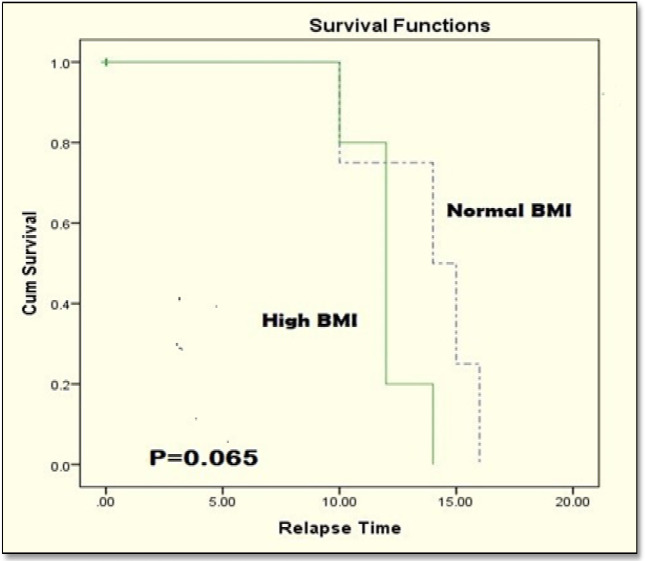
Disease-free Survival in the Studied Patients

**Figure 6:**
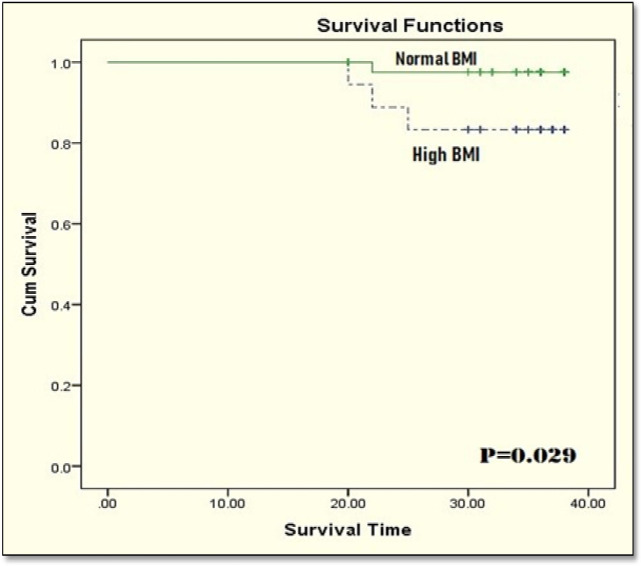
Overall Survival in the Studied Patients

Kaplan–Meyer survival analysis revealed no statistically significant differences between disease-free survival rates in normal and obese patients (log-rank p = 0.065) (Figure 5). However, patients with normal BMI had significantly better overall survival than patients who are obese (log-rank p = 0.029 (Figure 6).

## Discussion

Obesity is well-defined as “an excess accumulation of adipose tissue that occurs when energetic intake exceeds energy spending” (Heetun et al., 2017). Generally, body fat is normally distributed across the “visceral, subcutaneous, and internal tissues, mostly in the liver;” however, these normal patterns of distribution become disrupted in obese individuals (Palmer and Clegg, 2015). 

Obesity and overweight are usually considered as poor prognostic factors of early-stage breast cancer (Kogawa et al., 2015). Additionally, a high percentage of visceral fat diminishes the benefits of neo-adjuvant chemotherapy in breast cancer patients, particularly in postmenopausal women. 

Obese women with breast cancer represent a unique patient population. They are at an increased risk for development of breast cancer and may experience more complications related to surgery and radiation. Despite appropriate local disease treatment, obese women are also at an increased risk for local recurrence compared to normal-weight women. Similarly, systemic chemotherapy may be less effective, even when dosed appropriately according to actual weight. Additionally, endocrine therapy may be less effective in obese women, and there is a study suggesting that tamoxifen may be more effective than aromatase inhibitors in this population. Taken together, these data suggest a unique and aggressive biology that is likely due to a tumor environment metabolically activated by adipose tissues (Lee et al., 2019).

In the present study, the correlation between BMI and tumor grade (p = 0.76) and LVI (p = 0.42) was not statistically significant. This finding agrees with the results by Takahiro et al., (2018) for tumor grade (p = 0.5) and LVI (p = 0.92). Consequently, in the present study and that conducted by Takahiro and colleagues (2018), menopausal status was linked to BMI (p = 0.001 vs p < 0.001), as well as HER2 status (p = 0.04 vs p = 0.04) and pathology subtype (p = 0.037 vs p = 0.039). 

The findings reported by Ayoub et al., (2019) are similar to our results for menopausal status and BMI index (p < 0.001), disease stage (p = 0.24) and LVI (p = 0.45); however, there findings were contrary to those of ours regarding tumor grade (p = 0.003 for them Vs p= 0.76 for us) and pathology subtype (p = 0.191 for them Vs p = 0.037 for us). This may be attributable to the different sample sizes (348 patients); they also added a mixed lobular and ductal variant as the third group in comparison to the two groups used in our study. 

Although the association between obesity/overweight and the prognosis of breast cancer patients remains controversial, it has been proposed that the effect of BMI on prognosis of breast cancer status might be associated with the menopausal status (Warren et al., 2015). In a prospective cohort study including 512 women with early stage breast cancer (T1 -T3, N0-N1, and M0), increased BMI was strongly associated with poor DFS and OS (Yazici et al., 2015). 

In the present study, although there was no significant correlation between BMI and relapse rate (11.1% for normal group Vs 22.7 % for obese group, p = 0.16), BMI was statistically significantly correlated with disease-related mortality; hence, normal BMI patients had lower disease related-mortality rates than obese patients (2.3 % Vs 16.7 % respectively, p =0.023) (Table 2, Figure 1). 

Achieving pCR by neoadjuvant treatment has been associated with long-term clinical benefits, mostly in HER2-positive and triple-negative breast cancer (Colomer et al, 2019).

In the present study, the correlation between pCR and BMI was not statistically significant (p = 0.7), supporting the findings reported by Iwase et al., (2016) (chi-squared test; p = 0.88) and Sasanpour et al., (2018) (p= 0.84). These authors similarly reported the non-correlation between pCR and disease stage (chi-squared test; p = 0.12, p = 0.33 and = 0.21 respectively), and menopausal status (p = 0.27 in the present study vs p = 0.67 and p = 0.24 respectively). 

For the pCR correlation with pathology subtype (p = 0.61 with 93.8% IDC for our patients), we noticed a similar finding with that reported by Sasanpour et al., (2018) (p= 0.24 with IDC 95.5% for all patients) but contrary to that by Iwase et al., (2016) (p < 0.05., IDC were less than 90%). Additionally, HER2 status correlation was similar to the finding by Sasanpour et al., (2018) (p = 0.26 vs p =0.27). For hormone status, pCR is statistically significant in our study (p < 0.001) as identified with the results by Sasanpour et al., (2018) (p= 0.003). 

An assessment of 26 studies involving 29.460 women revealed that increased BMI was accompanied with adverse prognosis of breast cancer. In a meta-analysis, it was described that in patients with increased BMI, the recurrence risk was 1.91(95% CI, 1.52-2.40) at 5 years, and the mortality risk was 1.6 (95% CI, 1.38-1.76) at 10 years. These results suggest that obese women have increased risk of relapse and death (Yazici et al., 2015). 

Cox regression analysis showed that significant predictors of mortality in univariate analysis included HER2 receptors (HR: 0.03, CI: 0.0-76.9, p=0.034), estrogen receptors (HR: 39.1, CI: 4.7-326.0, P: 0.001), progesterone receptors (HR: 0.09, CI: 0.01- 0.7, P: 0.022), and Ki-67(HR: 1.06, CI: 1.02-1.1, p: 0.001). However, only estrogen receptors (HR: 16.1, CI: 0.9-281.5, P: 0.057) and Ki-67 (HR: 11.02, CI: 0.98-1.06, p: 0.38) variables were established in multi-variate analysis (Table 4). These results are consistence with finding by Kogawa et al., (2015) for HER2 and hormone receptors status. Sun et al., (2018) showed also the same finding with our study regarding ER by univariate analysis (p =.026) but not the same regarding ER in multivariate analysis (HR: 1.180, CI: 0.705–1.975, p = 528), this inconsistency may be due to different patients number (1017 pt. for them Vs 80 pts for us) and study designs ( three BMI groups in their study Vs two only in our study).

Finally, the limitations in our study include the retrospective design and the small sample size. These limitations may have contributed to discrepancies in our study, as we could not differentiate the study population according to subgroup analysis especially for the menopausal groups (principally in postmenopausal patients); further, we could not evaluate comorbidities, as obese patients are assumed to have higher risks of comorbid conditions. 

In Conclusion, in patients with early breast cancer treated with neo-adjuvant systemic therapy, high BMI/obesity is associated with lower pCR, higher relapse rates, and lower survival outcomes than normal BMI. Even though our study suggests a correlation between obesity, pCR, and breast cancer prognosis; further studies with larger sample sizes and more comprehensive designs are necessary. Additionally, the findings from this study can help in the development of standard health programs for the prevention of breast cancer. This can be done by improving patient orientation towards potentially changeable lifestyle risk factors such as obesity/overweight.

**Table 1:** Patients’ and Tumor Characteristics

Parameters	All patients N=80 (%)
Menopausal Status	
Premenopausal	16 (20.0)
Postmenopausal	64 (80.0)
BMI	
Normal	36 (45.0)
Obese	44 (55.0)
Disease stage	
I	21 (26.25)
II	26 (32.5)
IIIA	33 (41.25)
Histopathology	
IDC	75 (93.8)
ILC	5 (6.2)
ER Status	
Positive	67 (83.8)
Negative	13 (16.2)
PR Status	
Positive	51 (63.8)
Negative	29 (36.2)
HER2 Status	
Positive	17 (27.0)
Negative	63 (73.0)
Tumor grade	
Low	5 (6.25)
Intermediate	49 (61.25)
High	26 (32.5)
LVI	
Positive	26 (32.5)
Negative	54 ( 67.5)
pCR	
Yes	56 (70)
No	24 (30)
Ki-67 (%) median (IQR)*	20.0 (5.0-48.8)
Relapse	
Yes	14 (17.5)
No	66 (82.5)
Mortality	
Yes	7 (8.8)
No	73 (91.2)
Survival	
Disease free survival (months) median	14 (11.0 – 15.0)
Survival time (months) median	35.0 (34.0-36.0)

*IQR, interquartile range

**Table 2:** The Association between BMI and Patients’ Characteristics and Tumor Characteristics

	BMI n (%)		P value
	Normal BMI n=36	High BMI (Obese) n=44	
Menopausal Status			
Premenopausal =16pt.	13 (36.1)	3 (6.8)	0.001
Postmenopausal =64pt.	23 (63.9)	41 (93.2)	
Histopathology			
IDC =75 pt	36 (100.0)	39 (88.6)	0.037
ILC =5 pt	0 (0.00)	5 (11.4)	
Disease stage n (%)			
I = 21 pt	12 (33.3)	9 (20.5)	
II = 26 pt	11 (30.6)	15 (34.1)	0.5
IIIA = 33 pt	13 (36.1)	20 (45.4)	
ER status			
Positive =67 pt	26 (72.2)	41 (93.2)	0.011
Negative =13pt	10 (27.8)	3 (6.8)	
PR status			
Positive = 51 pt	24 (66.7)	27 (61.4)	0.62
Negative = 29 pt	12 (33.3)	17 (38.6)	
HER2			
Positive = 17 pt	4 (11.1)	13 (29.5)	0.045
Negative = 63 pt	32 (88.9)	31 (70.5)	
Tumor grade n (%)			
Low = 5 pt	3 (8.3)	2 (45.5)	0.76
Intermediate = 49 pt	21 (58.3)	28 (36.7)	
High = 26 pt	12 (33.3)	14 (31.8)	
LVI n (%)	10 (27.8)	16 (36.4)	0.42
Ki-67 (%) median (IQR)	15.0 (5.0 – 45.0)	35 (7.5 – 75.0)	0.11
Disease status/Treatment outcome			
Relapse n (%)	4 (11.1)	10 (22.7)	0.16
Relapse time (months)	14.5	12.0	0.17
Median (IQR)	(11.0 – 15.8)	(11.0 - 13.0)	
Survival time (months)	35.0	35.5	0.15
Median (IQR)	(31.0 – 37.0)	(34.0-36.0)	
Mortality n (%)	1 (2.3)	6 (16.7)	0.023

**Table 3:** Comparison between Patients with pCR and Patients without Regarding the Clinical Data

		pCR		P- value
		Yes n=56	No n=24	
Menpausal				
Premenopausal =16pt.		13 (30.2)	3 (12.5)	0.27
Postmenopausal =64pt.		43 (69.8)	21 (87.5)	
BMI				
Normal BMI (Up to 25)= 36 pt.		26 (46.4)	10 (41.7)	0.7
High BMI(more than 25) = 44pt		30 (53.6)	14 (58.3)	
Histopathology				
IDC =75 pt		53 (94.6)	22 (91.7)	0.61
ILC =5 pt		3 (5.4)	2 (8.3)	
HER2 n (%)				
Positive = 17 pt		10 (17.9)	7 (29.2)	0.26
Negative = 63 pt		46 (82.1)	17 (70.8)	
ER status				
Positive =67 pt		56 (100.0)	11 (45.8)	< 0.001
Negative =13 pt		-	13 (56.2)	
PR status				
Positive = 51pt		50 (89.3)	1 (4.2)	< 0.001
Negative = 29 pt		6 (10.7)	23 (95.8)	
Tumor grade n (%)				
Low = 5 pt		5 (8.9)	0 (0.00)	0.004
Intermediate = 49 pt		38 (67.9)	11 (45.8)	
High = 26 pt		13 (23.2)	13 (54.2)	
Disease stage n (%)				
I = 21 pt		16 (28.6)	5 (20.8)	0.12
II = 26 pt		21 (37.5)	5 (20.8)	
IIIA = 33 pt		19 (33.9)	14 (58.4)	
Ki-67 (%) median	20.0 (5.0-48.8)	15.0 (2.3-40.0)	70.0 (30.0 – 80.0)	< 0.001
LVI n (%)	26 (32.5)	15 (26.8)	11 (45.8)	0.1
Disease status /Treatment outcome				
Relapse	14(17.5)	2(3.57)	12 (50.0)	< 0.001
Relapse free survival (months) median (IQR)	14 (11.0 – 15.0)	10	14 (12-15)	0.17
Survival time (months) median (IQR)	35.0 (34.0-36.0)	36.0 (35.0-37.0)	31.0 (22.0-36.0)	< 0.001
Mortality	7 (8.8)	1 (1.8)	6 (25.0)	0.001

**Table 4:** Predictors of Survival in the Studied Patients

Univariate analysis			
	HR	95% CI	P
Menopausal			
Pre- Vs Postmenopausal.	0.67	0.1 – 3.5	0.64
BMI			
Normal Vs Obese	0.14	0.02-1.1	0.065
HER2			
Negative Vs Positive	0.03	0.0-76.9	0.034
Estrogen receptors n (%)			
Positive Vs Negative	39.1	4.7-326.0	0.001
Progesterone receptors n (%)			
Positive Vs Negative	0.09	0.01-0.7	0.022
Ki-67	1.06	1.02-1.1	0.001
Multivariate analysis			
	HR	95% CI	P
Estrogen receptors n (%)			
Positive Vs Negative	16.1	0.9-281.5	0.057
Ki-67	1.02	0.98-1.06	0.38
